# Small-Cell Neuroendocrine Cervical Carcinoma Mimicking a Polyp in a 20-Year-Old Patient With a Five-Year Follow-Up: A Case Report

**DOI:** 10.7759/cureus.62893

**Published:** 2024-06-22

**Authors:** Chau Giang Huynh, Bich-Ha Thi Truong, Truc Thanh Thai, Phuong-Thao Thi Doan

**Affiliations:** 1 Department of Pathology, Hung Vuong Hospital, Ho Chi Minh City, VNM; 2 Department of Obstetrics and Gynecology, Pham Ngoc Thach University of Medicine, Ho Chi Minh City, VNM; 3 Department of Medical Statistics and Informatics, Faculty of Public Health, University of Medicine and Pharmacy at Ho Chi Minh City, Ho Chi Minh City, VNM; 4 Department of Pathology, University of Medicine and Pharmacy at Ho Chi Minh City, Ho Chi Minh City, VNM

**Keywords:** small cell neuroendocrine carcinoma, case report, immunohistochemistry, neuroendocrine tumors, cervical cancer

## Abstract

Small-cell neuroendocrine cervical carcinoma (NECC) is a rare histology, and diagnosis and treatment of this condition are challenging because of its rarity, non-specific abdominopelvic symptoms, and less favorable prognosis compared to other cervical cancers. Here, we present a case of a 20-year-old patient diagnosed with small-cell NECC, defined within a cervical polyp, initially mimicking a benign lesion. Because of the difficulty in diagnosis, the patient underwent thorough diagnostics and interventions, including imaging, histopathology, and immunohistochemistry. Initially, the patient underwent a distinctive treatment plan encompassing four cycles of cisplatin and etoposide chemotherapy with minimal side effects. Subsequently, she received comprehensive surgical interventions, including hysterectomy, lymphadenectomy, and bilateral salpingectomy. Ovarian preservation, justified by the patient’s youth and small cervical lesions (<4 cm) without parametrial disease or metastatic signs, was pursued. For long-term outcomes, the patient demonstrated no metastasis or recurrence during a five-year follow-up. This case emphasizes the requirement for strengthened awareness of neuroendocrine tumors in cervical masses, particularly in young patients, and the importance of individualized treatment approaches for optimal clinical outcomes. Continued documentation of such cases increases our understanding of managing infrequent cervical malignancies.

## Introduction

Small-cell neuroendocrine cervical carcinoma (NECC), a rare condition, represents less than 2% of all invasive cervical cancers [1, 2]. According to the Surveillance, Epidemiology, and End Results database, the mean annual incidence in the United States from 1977 to 2003 was 0.06 per 100,000 women, significantly lower than the rates for squamous cell carcinoma and adenocarcinoma of 6.6 and 1.2 per 100,000 women, respectively [2]. Diagnosing NECC is often difficult because patients typically present with atypically abdominopelvic symptoms such as vaginal bleeding or discharge, pelvic pain, or pelvic pressure, which can be observed in many benign conditions. Very few patients are asymptomatic or present after an abnormal Pap smear result [3].

NECC is indistinguishable from pulmonary small-cell neuroendocrine carcinoma and extrapulmonary small-cell carcinoma of other sites. Therefore, all patients should undergo a computed tomography of the chest, abdomen, and pelvis to assess the extent of locoregional disease and distant spread [4]. A prevailing approach among clinicians involves the use of combined modality therapy (surgery followed by chemotherapy or combined chemoradiotherapy) [4] for limited-stage potentially resectable disease, definitive chemoradiotherapy for locoregionally advanced unresectable but nonmetastatic disease, and palliative chemotherapy alone for those with metastatic disease. The chemotherapy regimens employed in such cases are typically those used for small-cell lung cancer [4].

Patients diagnosed with small-cell NECC have a less favorable prognosis compared to those with cervical squamous cell carcinomas or adenocarcinomas [2], although it is relatively better than the prognosis for small-cell lung cancer. The five-year survival rate is approximately 30% for those with limited-stage disease, but the prognosis is grim for patients with more extensive disease, with few surviving beyond two years. Key adverse prognostic factors include advanced tumor stage, large tumor size, pure small-cell histology, and a history of smoking [5, 6]. Small-cell NECC exhibit a higher likelihood of lymph node metastases and lymphovascular space invasion, and their clinical course is characterized by early hematogenous dissemination [3, 7].

In this report, we present a case of a very young patient who had a lesion within a polyp in the cervix, raising the risk of misdiagnosis as a benign condition. The patient was finally diagnosed with small-cell NECC and underwent the conventional modality therapy with a 63-month follow-up.

## Case presentation

**Figure 1:**
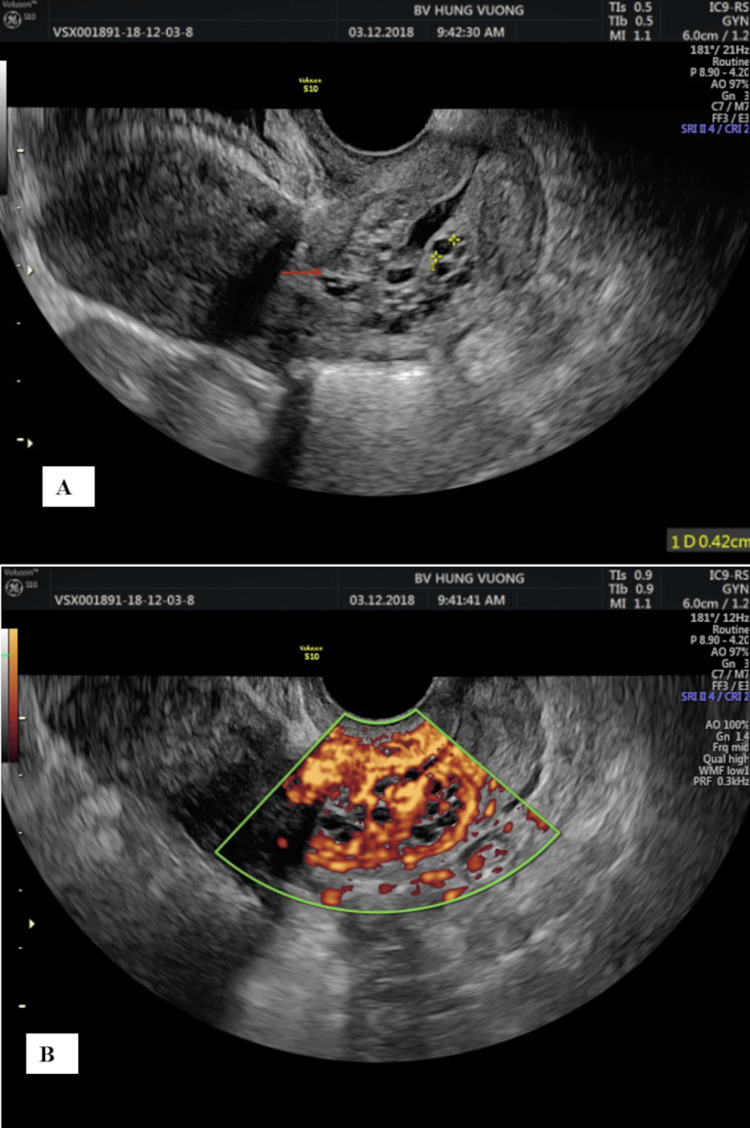
Lesions present in the cervix on 2-D ultrasound images (A) and color ultrasound images (B)

A 20-year-old patient with a G1P100 history was transferred to our hospital from another private clinic due to recent findings of a large endocervical polyp and a positive HPV18 test (Table 1). The patients received no interventions at that clinic because it was beyond their professional capabilities. Upon arrival, the patient did not exhibit any abdominopelvic symptoms such as vaginal bleeding, discharge, or pelvic pain. She began sexual activity at the age of 14. The patient was not vaccinated for HPV and had only one sexual partner. The external examination of the vulva and vagina found a perineal scar from a previous episiotomy, with no notable signs of tumors or ulcers. The speculum examination identified a 2x2x3 cm mass, slightly extruding out of the endocervix. Afterward, ultrasound imaging confirmed a lesion with many blood vessels inside the posterior cervical lip, which was 2x2x3 cm in size. The ultrasound also showed normal results in the uterus, ovarian tubes, and ovaries (Figure 1). The patient was initially diagnosed with a large cervical polyp and underwent a simple cervical polypectomy to remove it. Based on its appearance and characteristics, the cervical mass appeared consistent with a cervical polyp during the initial clinical examination. However, the mass was larger and firmer during the operative procedure than typical polyps, with a smooth, rubbery surface. It was attached via a broad base to the muscular layer, a common feature of leiomyomas. These findings made the physicians suspect it was a leiomyoma rather than a polyp.

**Table 1:** Patient’s health and medication history

Characteristics	Value
Body mass index	18.8 kg/m²
HPV high risk	Type 18
Blood Cell Counts	
White blood cells (WBC)	7.2 K/µL
Hemoglobin	91.70 g/L
Platelets	195 K/µL
Vital Organ Function Tests	
Liver function (ALT, AST)	ALT: 15.6 U/L, AST: 6.5 U/L
Kidney function (creatinine, BUN)	Creatinine: 83.8 µmol/L, Ure: 5.3 mmol/L
CA125	21.56 UI/mL
HIV screening	Negative
HBV screening	Negative
HCV screening	Negative
Medications	
Chemotherapy regimen	Cisplatin and etoposide
Dosage adjustments	Adjusted for low BMI
Follow-up Results	
Latest follow-up (5 years)	No signs of metastatic or recurrent disease
Clinical observation	Normal eating and drinking habits, returned to work

**Figure 2:**
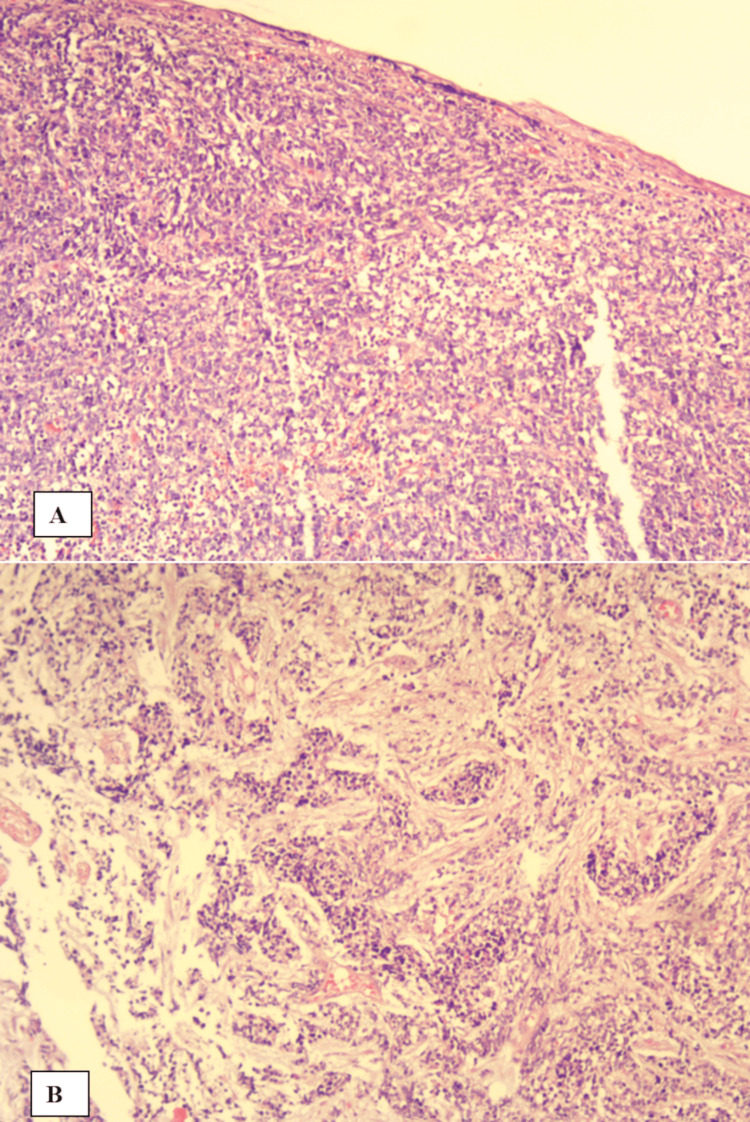
(H&E, x10) Poorly differentiated, diffusely infiltrating small tumor cells (A) and neoplastic cells typically arranged in clusters, sheets, or trabeculae, separated by a delicate fibrovascular stroma (B)

**Figure 3:**
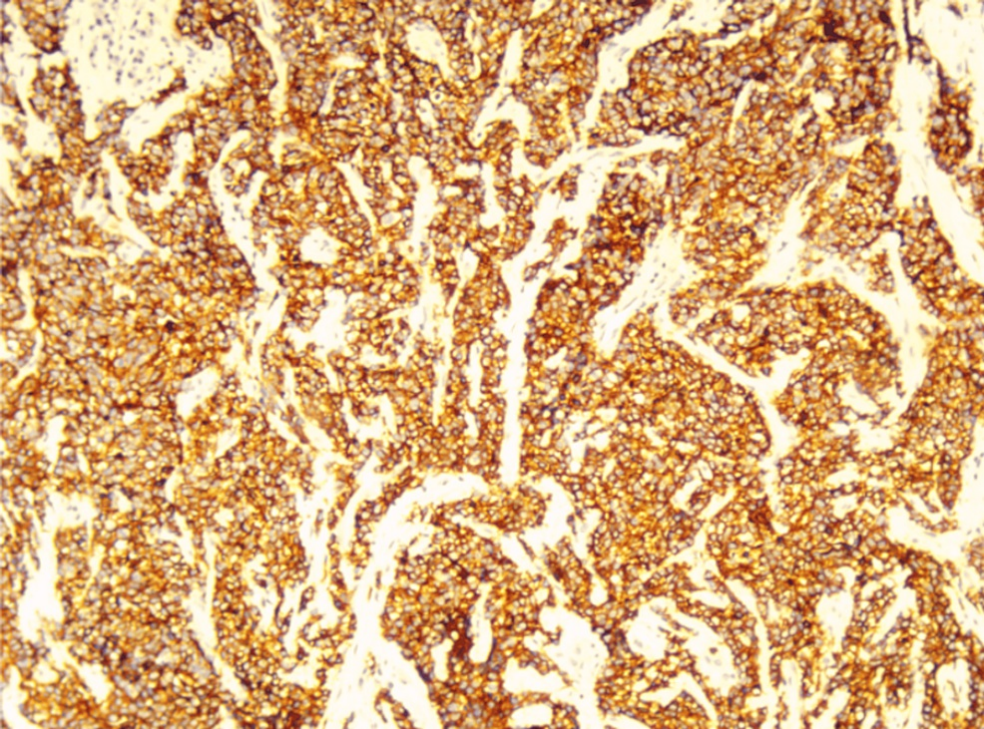
Positive results for CD56 staining in neuroendocrine cells in immunohistochemistry

Under microscopic examination using hematoxylin-eosin staining, the sections revealed poorly differentiated, diffusely infiltrating small tumor cells. These cells had scant cytoplasm, hyperchromatic nuclei with finely dispersed chromatin, and absent or inconspicuous nucleoli. Neoplastic cells were arranged in clusters, sheets, or trabeculae, separated by a delicate fibrovascular stroma infiltrated by chronic inflammatory cells such as plasma cells and lymphocytes. Mitotic figures were high, and necrosis of individual tumor cells was observed (Figure 2). The surgical margin of the lesion no longer showed malignant tumor cells. Immunohistochemical evaluation included staining for CD56 to confirm the diagnosis (Figure 3). Post-diagnosis evaluation, including chest X-ray and CT scans of the chest, abdomen, and pelvis, was performed to evaluate the extent of the disease. The results confirmed no secondary lesions in other sites. Given the tumor size (i.e., <4 cm) and the absence of evidence of lymph nodes and metastases in other sites, the final diagnosis was a high-grade neuroendocrine tumor, small-cell type, classified as T1b2N0M0, FIGO stage IB2.

The patient was discussed with a multidisciplinary tumor board consisting of oncologists and gynecologists. Given the patient’s young age, we discussed options for trachelectomy or uterine preservation after chemotherapy to maintain the patient’s reproductive viability. The initial plan was to perform a hysterectomy after the patient had the desired number of children. The patient was thoroughly informed about each treatment option’s potential risks and benefits. After being explained and understanding how to treat this rare condition, however, the patient chose to receive chemotherapy first and then surgery. Then, the patient received a four-cycle regimen of chemotherapy consisting of cisplatin (20 mg/m^2^) and etoposide (100 mg/m^2^). Each cycle occurred at 21-day intervals, with concurrent infusion of both drugs from day one to day five. Hospitalization for seven days followed each drug cycle to monitor side effects. Throughout the treatment, the patient experienced minor side effects, including nausea on the second day and vomiting on the fourth and fifth days. Despite these side effects, electrolyte levels remained within normal limits for each cycle. In cycles three and four, there was a slight decrease in red and white blood cell counts, but liver and kidney function remained normal. The patient, however, exhibited weight loss from 45 kg to 42 kg, leading to a dosage adjustment in cycles three and four. The body skin area decreased from 1.41 m^2^ in cycles one and two to 1.35 m^2^ in cycles three and four. Subsequently, she underwent radical hysterectomy (hysterectomy, fallopian tube resection, pelvic lymph node dissection). The decision to preserve the patient’s ovaries was based on her youth and the presence of small (<4 cm) cervical lesions, with no observable parametrial disease or signs of metastatic spread.

One week post-operation, the patient was in good health and subsequently discharged. Her final pathology from her surgery had no malignant tumor cells. A follow-up examination occurred one month later, and post-treatment surveillance followed a detailed schedule in this case. Specifically, the patient underwent monthly examinations for the first nine months, then five follow-up examinations every three months, and five follow-up visits every six months. One month after surgery, the patient demonstrated clinical improvement, including normal eating and drinking habits and returned to work. As of the most recent follow-up, approximately five years after surgery, the patient’s overal health was still good. There were no signs of widespread metastatic or recurrent disease, with normal screening tests and liver and kidney function within normal limits.

## Discussion

In ≤20-year-old patients, malignant diseases are rare, particularly small-cell neuroendocrine carcinoma of the cervix (NECC). This contrasts with the findings of Gardner et al. where the typical age range for patients was from 21-87 years, with a median age of 50 years [4]. Therefore, many patients at this age can be initially misdiagnosed since most clinicians infrequently consider the chance of malignancy at this age, despite the patient having a risk factor for cervical cancer, such as high-risk HPV type 18 infection. Furthermore, manifestations of the lesion often lack specificity, and the clinical features, along with the preferred site for small-cell neuroendocrine carcinoma, closely resemble those observed in cervical cancer patients. However, it is essential to note that small-cell NECC is not typically associated with HPV [4].

Diagnosing small-cell NECC is challenging because it closely resembles small-cell carcinoma of the lung and extrapulmonary small-cell carcinoma in other locations. The diagnosis for our patient also required both tissue biopsy for routine H&E staining and immunohistochemistry targeting neuroendocrine markers as a standard guideline [8]. We conducted a comprehensive systemic imaging assessment covering the chest, abdomen, and pelvis to eradicate the possibility of metastasis from other sites. This evaluation was essential due to the increased incidence of lymphatic and vascular invasion and distant organ metastases, typically observed in patients with early-stage small-cell NECC, leading to a less favorable prognosis [4]. Despite these potential risks, our patient presented no indications of lymph node involvement or metastases in other sites. As a result, the final diagnosis confirmed a high-grade neuroendocrine tumor of the small-cell type, categorized as T1b2N0M0, FIGO stage IB2 [9].

The optimal treatment strategy for small-cell NECC remains controversial. While most clinicians typically advocate for a combined modality therapy approach involving surgery followed by chemotherapy or combined chemoradiotherapy [4], our patient received a distinctive treatment plan. The detailed treatment plan was thoroughly discussed with the patient, who demonstrated clear understanding and provided informed consent for the proposed interventions. Our patient underwent a four-cycle cisplatin and etoposide chemotherapy regimen, similar to the recommended treatment plan in the literature [4, 10]. However, we adjusted the chemical dosage to match the patient’s constitution, as her BMI was only 18.8 kg/m². Typically, the suggested regimen involves a single dose of cisplatin on day one at 80 mg/m^2^ of skin and etoposide at 100 mg/m^2^ of skin on days one to three of each cycle. Our patient received simultaneous cisplatin and etoposide for five days per cycle, with different dosages from the recommendations (cisplatin 20 mg/m2; etoposide 100 mg/m^2^). Therefore, our patient experienced only mild side effects and resumed normal eating and drinking habits after one week of each chemotherapy cycle.

The role of surgery in small-cell neuroendocrine carcinoma remains controversial due to its rarity and limited research. For our patient, because of the absence of clear parametrial disease and no evidence of metastatic disease, the choice of surgical intervention (consisting of hysterectomy, fallopian tube resection, and lymphadenectomy) after chemotherapy is an appropriate option, especially for patients with small (<4 cm) cervical lesions. Our approach is consistent with guidance from the Society of Gynecologic Oncology [4]. Furthermore, findings from a previous study involving 188 patients underscored the significance of radical hysterectomy as an independent prognostic factor for survival in multivariate analysis, in conjunction with early-stage disease and the utilization of any chemotherapy. Notably, among patients diagnosed with stage I to IIA disease, those who underwent radical hysterectomy exhibited a significantly higher five-year survival rate compared to those who did not undergo the procedure (38% versus 24%) [5].

In terms of treatment effectiveness, early detection is an essential key to achieving disease-free survival for the patient. Although women diagnosed with small-cell NECC face a comparatively less favorable prognosis when contrasted with cervical squamous cell carcinomas or adenocarcinomas, research indicates that primary favorable prognostic factors include early tumor stage, tumor size <4 cm, and no clinical signs of nodal metastases, demonstrating a better prognosis [8, 11]. Moreover, we decided to perform monthly follow-ups at the beginning rather than following the classical cervical cancer protocol because of the aggressive nature of small-cell NECC. NECC is known for its rapid progression and high recurrence rate, necessitating more frequent monitoring to detect any early signs of recurrence or metastasis. This intensive follow-up schedule can help facilitate timely interventions, potentially improving the patient’s prognosis and outcome. Our patient, monitored over a five-year follow-up period, continued to have good health with no signs of widespread metastasis or recurrent disease, further supporting these prognostic observations.

## Conclusions

This case report emphasizes the complexities in diagnosing and treating small-cell NECC, particularly in young patients. The infrequency of this malignancy, along with its potential to mimic benign conditions like cervical polyps, highlights the critical need for intensified attention among clinicians, even in relatively young patients. The case also underscores the successful application of a personalized treatment approach leading to favorable clinical outcomes. Ongoing research and documentation of similar cases significantly contribute to increasing our comprehension of the optimal management strategies for uncommon cervical malignancies.

## References

[1] Albores-Saavedra J, Gersell D, Gilks CB (1997). Terminology of endocrine tumors of the uterine cervix: results of a workshop sponsored by the College of American Pathologists and the National Cancer Institute. Arch Pathol Lab Med.

[2] Chen J, Macdonald OK, Gaffney DK (2008). Incidence, mortality, and prognostic factors of small cell carcinoma of the cervix. Obstet Gynecol.

[3] Cohen JG, Kapp DS, Shin JY (2010). Small cell carcinoma of the cervix: treatment and survival outcomes of 188 patients. Am J Obstet Gynecol.

[4] Gardner GJ, Reidy-Lagunes D, Gehrig PA (2011). Neuroendocrine tumors of the gynecologic tract: A Society of Gynecologic Oncology (SGO) clinical document. Gynecol Oncol.

[5] Chan JK, Loizzi V, Burger RA, Rutgers J, Monk BJ (2003). Prognostic factors in neuroendocrine small cell cervical carcinoma: a multivariate analysis. Cancer.

[6] Videtic GM, Stitt LW, Dar AR (2003). Continued cigarette smoking by patients receiving concurrent chemoradiotherapy for limited-stage small-cell lung cancer is associated with decreased survival. J Clin Oncol.

[7] Sevin BU, Lu Y, Bloch DA, Nadji M, Koechli OR, Averette HE (1996). Surgically defined prognostic parameters in patients with early cervical carcinoma. A multivariate survival tree analysis. Cancer.

[8] Viswanathan AN, Deavers MT, Jhingran A, Ramirez PT, Levenback C, Eifel PJ (2004). Small cell neuroendocrine carcinoma of the cervix: outcome and patterns of recurrence. Gynecol Oncol.

[9] Bhatla N, Berek JS, Cuello Fredes M (2019). Revised FIGO staging for carcinoma of the cervix uteri. Int J Gynaecol Obstet.

[10] Alfsen GC, Thoresen SO, Kristensen GB, Skovlund E, Abeler VM (2000). Histopathologic subtyping of cervical adenocarcinoma reveals increasing incidence rates of endometrioid tumors in all age groups: a population based study with review of all nonsquamous cervical carcinomas in Norway from 1966 to 1970, 1976 to 1980, and 1986 to 1990. Cancer.

[11] McCusker ME, Coté TR, Clegg LX, Tavassoli FJ (2003). Endocrine tumors of the uterine cervix: incidence, demographics, and survival with comparison to squamous cell carcinoma. Gynecol Oncol.

